# Dr. M'Divit

**Published:** 1840-01

**Authors:** 


### JOHN M'DIVIT, M.D., OF CANTERBURY.

Died on the 14th instant, at Bishopsbourne, Kent, at an early age, Dr. M'Divit, one of the physicians of the Kent and Canterbury hospital. He was the author of several valuable communications in our journals, which gave promise of more important contributions to medical science, had his valuable life been spared.

